# A Genetic Strategy to Measure Circulating *Drosophila* Insulin Reveals Genes Regulating Insulin Production and Secretion

**DOI:** 10.1371/journal.pgen.1004555

**Published:** 2014-08-07

**Authors:** Sangbin Park, Ronald W. Alfa, Sydni M. Topper, Grace E. S. Kim, Lutz Kockel, Seung K. Kim

**Affiliations:** 1 Department of Developmental Biology, Stanford University School of Medicine, Stanford, California, United States of America; 2 Neuroscience Program, Stanford University School of Medicine, Stanford, California, United States of America; 3 Department of Medicine (Oncology Division) Stanford University School of Medicine, Stanford, California, United States of America; 4 Howard Hughes Medical Institute, Stanford University School of Medicine, Stanford, California, United States of America; Washington University Medical School, United States of America

## Abstract

Insulin is a major regulator of metabolism in metazoans, including the fruit fly *Drosophila melanogaster*. Genome-wide association studies (GWAS) suggest a genetic basis for reductions of both insulin sensitivity and insulin secretion, phenotypes commonly observed in humans with type 2 diabetes mellitus (T2DM). To identify molecular functions of genes linked to T2DM risk, we developed a genetic tool to measure insulin-like peptide 2 (*Ilp2*) levels in *Drosophila*, a model organism with superb experimental genetics. Our system permitted sensitive quantification of circulating Ilp2, including measures of Ilp2 dynamics during fasting and re-feeding, and demonstration of adaptive Ilp2 secretion in response to insulin receptor haploinsufficiency. Tissue specific dissection of this reduced insulin signaling phenotype revealed a critical role for insulin signaling in specific peripheral tissues. Knockdown of the *Drosophila* orthologues of human T2DM risk genes, including *GLIS3* and *BCL11A*, revealed roles of these *Drosophila* genes in Ilp2 production or secretion. Discovery of *Drosophila* mechanisms and regulators controlling *in vivo* insulin dynamics should accelerate functional dissection of diabetes genetics.

## Author Summary

Genome-wide association studies in patients with type 2 diabetes mellitus have identified more than 65 loci, encoding up to 500 candidate susceptibility genes. Thus, investigators are fundamentally challenged to (i) screen and identify relevant candidates *in vivo*, (ii) determine if loss- or gain-of-function underlies the association, (iii) link perturbed gene function to hallmark type 2 diabetes mellitus physiological phenotypes like insulin production or secretion, and (iv) identify relevant tissue(s) where the biological function of a specific regulator is required. Here we exploit *Drosophila* genetics to reveal the molecular functions of evolutionally conserved regulators that are associated with human type 2 diabetes mellitus. Targeted knockdown of *Drosophila* orthologues of diabetes risk genes revealed tissue-specific roles for these genes in regulating insulin production and secretion. These findings should accelerate use of *Drosophila* and other genetically-tractable systems to discover conserved mechanisms and regulators controlling *in vivo* insulin dynamics relevant to diabetes and other human diseases.

## Introduction

Insulin is a major regulator of metabolism, growth and development in metazoans, including the fruit fly *Drosophila melanogaster*. Insulin resistance in the liver and other human tissues can lead to compensatory increases in insulin production and secretion by pancreatic β cells, a facultative response that fails during pathogenesis of type 2 diabetes mellitus (T2DM) [1]. The decline of both insulin sensitivity and insulin secretion may have a genetic basis in humans [2]. *Drosophila* could emerge as a powerful system for dissecting the genetics of insulin resistance and secretion if appropriate physiological assays, like quantification of circulating insulin, could be used to assess insulin dynamics.

*Drosophila* Insulin-like peptide 2, 3 and 5 (Ilp2, 3, and 5) are synthesized and secreted by insulin producing cells (IPCs), median neurosecretory cells located in the pars intercerebralis, and are crucial hormonal regulators of development, growth and metabolism [3], [4]. Ilp2 is a principal circulating insulin in flies, and is essential for maintaining normoglycemia [5]. Structural and biochemical studies of *Drosophila* insulin-like peptide association with its receptor suggest that Ilps might circulate at picomolar levels, similar to mammals [6]. Other than in mammals, however, no methods for determining the absolute concentration of circulating insulin with picomolar sensitivity exist, to our knowledge. The most widely-used method for assessing insulin secretion by *Drosophila* IPCs involves estimating the immuno-reactive signal for Ilp2 in IPCs [7]. By comparing the relative intensity of signal between experimental and control conditions, increased signal has been interpreted to indicate reduced or impaired secretion of insulin from IPCs. However, in using intracellular Ilp2 immunoreactivity as a surrogate for secretion, this method does not differentiate between changes in insulin production and secretion. To overcome these challenges, focus has shifted to the use of enzyme-linked immunosorbent assay (ELISA) as a potential method for Ilp2 quantification. An immunoepitope tagged Ilp2 was used to measure circulating Ilp2 by ELISA [8], but the tagged Ilp2 was overexpressed in IPCs, making it difficult to assess physiological regulation of Ilp2 production and secretion. A recent study used polyclonal antibodies to measure circulating Ilp2 and Ilp5 in adult hemolymph by ELISA [9], but only relative changes were reported. Moreover, the specialized nature of required reagents, like synthetic Ilp standards, have limited widespread adoption of this assay. Polypeptide-based immunoepitope tags can facilitate ELISA construction, but prior attempts over several decades to epitope-tag insulin, which undergoes extensive post-translational modification [6], have led invariably to loss or elimination of bioactivity [10], [11]. Maintenance of bioactivity in an epitope-tagged insulin would ensure that native mechanisms controlling crucial elements of insulin biology, like processing, storage, secretion and clearance, are being assayed. However, the bioactivity of prior epitope-tagged forms of Ilp2 has not been demonstrated or quantified [12].

Here we report the successful labeling of Ilp2, a crucial regulator of glucose metabolism in *Drosophila*, with two immuno-epitopes at specific positions that preserved Ilp2 bioactivity. Using unique fly strains expressing double-tagged Ilp2 (Ilp2HF), we developed robust and sensitive ELISA methods for quantifying circulating Ilp2 at picomolar concentration in *Drosophila*, and show that only a small fraction of total Ilp2 is secreted from IPCs *in vivo* and *in vitro*, like in mammals. Our studies reveal changes in either Ilp2 expression or secretion resulting from IPC-specific knockdown of *Drosophila* cognates of human T2DM genome-wide association study (GWAS) candidate loci, demonstrating genetic and molecular mechanisms linking these risk genes to insulin regulation. In addition, we uncovered previously undetected forms of genetic insulin receptor haploinsufficiency accompanied by adaptive insulin hypersecretion. Tissue specific dissection of this phenotype revealed a critical role for insulin signaling in the fat body in feedback regulation of systemic insulin levels. Thus, we provide the community with a potent new *Drosophila* tool for studies of insulin biology, integrative physiology and the genetic basis of human metabolic diseases.

## Results

### Synthetic lethality test of epitope-tagged *Drosophila* Insulin-like peptide 2 variants

**Figure 1:**
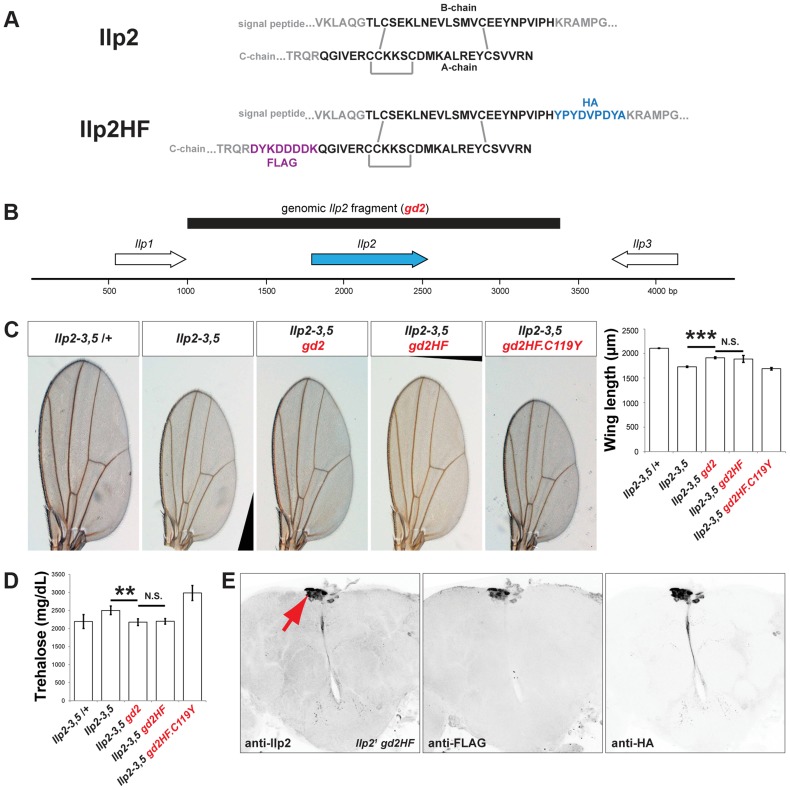
Epitope-tagged Ilp2 rescues insulin deficiency phenotypes. (A) Prepro-Ilp2 peptide sequence and locations of HA (blue) and FLAG (purple) epitopes inserted in Ilp2HF sequence. The sequences in black represent the putative mature Ilp2. Gray bars indicate conserved cysteine bonds in insulin-like peptides. (B) 2.4 kilobase pair genomic fragment (black bar) containing *Ilp2* gene and its regulatory sequence. (C) Quantification of wing length in flies lacking *Ilp2*, *Ilp3* and *Ilp5* genes (*Ilp2–3, 5*) with or without *gd2*, *gd2HF*, and *gd2HF.C119Y* genomic rescue fragments, as indicated. (D) Measurement of hemolymph trehalose concentration in insulin deficient flies with or without *gd2* or *gd2HF*, and *gd2HF.C119Y*. (E) Expression of *Ilp2HF* in *Ilp2^1^ gd2HF* adult insulin producing neurons (arrow) detected by anti-Ilp2, anti-FLAG, and anti-HA antibodies. In all figures, center values are averages, error bars represent the standard deviation, and two-tailed *t*-tests were used to generate *p* values. * indicates *p*<0.05, ** *p*<0.01, and *** *p*<0.001. N.S. indicates statistically not significant.

To measure a circulating *Drosophila* insulin directly, we sought to tag *Drosophila* Insulin-like peptide 2 (*Ilp2*) with immuno-detectable epitopes while preserving its *in vivo* bioactivity. Broad misexpression of a transgene encoding *Ilp2* from GAL4-responsive upstream activation sequences (UAS) is lethal [13], providing an assay of Ilp2 activity *in vivo*. To screen for permissive epitope-insertion sites that preserved the bioactivity of Ilp2, we misexpressed transgenes encoding variant forms of hemagglutinin- (HA) and FLAG-epitope-tagged Ilp2 (Figure S1), and scored the resulting lethality. Like mammalian insulins, *Drosophila* Ilp2 is comprised of ‘B-chain’ and ‘A-chain’ polypeptides linked by disulfide bonds (Figure 1A). Systematic variation of epitope position in the B-chain and A-chain of Ilp2 led to identification of tagged forms that remained lethal when broadly expressed, including a variant with HA-epitope fused to the B-chain carboxy-terminus and FLAG-epitope fused to the A-chain amino terminus (hereafter called “Ilp2HF”; Figure 1A). In contrast, a previously described FLAG-epitope labeled Ilp2 [12] achieved only 38% lethality (Figure S1). Substitution of a conserved A-chain cysteine by tyrosine (from a missense mutation called ‘Akita’) impairs insulin processing and activity in rodents [14], [15], and the orthologous substitution (C119Y) prevented Ilp2HF-induced lethality (Figure S1). Thus, we identified epitope-tags and positions in Ilp2 that preserved *in vivo* bioactivity in a synthetic lethality screen.

### Epitope-tagged Ilp2 rescues insulin deficiency phenotypes

To assess if Ilp2HF also retained native Ilp2 function and activity in regulating development, growth, and metabolism, we generated a 2.4 kilobase pair genomic fragment (Figure 1B) containing the native *Ilp2* or *Ilp2HF* gene under the control of the endogenous *Ilp2* regulatory sequence (*gd2* and *gd2HF*, respectively). We next assessed if developmental, growth, and metabolic defects observed in flies lacking *Ilp2*, *Ilp3*, and *Ilp5* (*Ilp2–3, 5*) [5] could be rescued by introducing *gd2* or *gd2HF* into *Ilp2–3, 5* mutants. Development from egg to adult eclosion in *Ilp2–3, 5* mutant females requires an average of 16 days, compared to 10 days for control flies (Figure S2). The delay is shortened to 11 days in mutants harboring a genomic *Ilp2* rescue construct (*Ilp2–3, 5 gd2*) and 12 days in mutants harboring a genomic *Ilp2HF* rescue construct (*Ilp2–3, 5 gd2HF*; Figure S2). Thus, both *gd2* and *gd2HF* substantially rescued the developmental delay observed in insulin deficient flies, although the eclosion time was delayed by 1–2 days with *gd2HF* rescue. In addition, both *gd2* and *gd2HF* rescued the reduced wing length of *Ilp2–3, 5* mutant adult flies to the same degree (Figure 1C). In *Ilp2–3, 5* mutant flies, elevated levels of trehalose, the major circulating form of sugar in flies, were also rescued by either *gd2* or *gd2HF* (Figure 1D). However, the C119Y missense mutant form of *gd2HF* failed to rescue developmental delay, wing length or trehalose phenotypes in *Ilp2–3, 5* flies (Figures 1C and 1D, Figure S2). Thus, Ilp2HF rescues severe insulin-deficiency to an extent comparable to native Ilp2, providing a unique example of a dual epitope-tagged insulin that retains *in vivo* biological activity that is nearly indistinguishable from native insulin.

### Physiological regulation of Ilp2HF production and secretion

**Figure 2:**
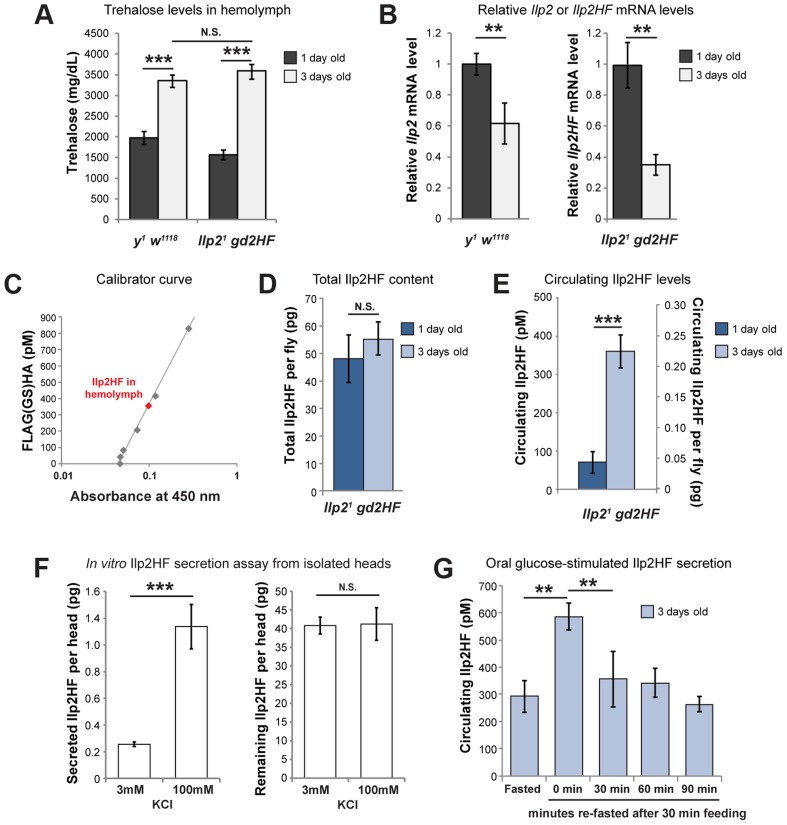
Physiological regulation of Ilp2HF production and secretion. (A) hemolymph trehalose concentrations in 1 and 3 day-old *y^1^ w^1118^* control and *Ilp2^1^ gd2HF* flies. (B) *Ilp2* or *Ilp2HF* mRNA levels in 1 and 3 day-old *y^1^ w^1118^* control and *Ilp2^1^ gd2HF* flies. (C) Circulating Ilp2HF concentration in hemolymph was determined by sandwich ELISA using 41 pM (0.1 pg/µl) to 4150 pM (10 pg/µl) of FLAG(GS)HA, a peptide harboring FLAG and HA epitopes, but up to 830 pM are shown in the curve. (D) Measurement of the total Ilp2HF content in 1 and 3 day-old *Ilp2^1^ gd2HF* flies. (E) Circulating Ilp2HF concentration (pM) or content per fly (pg) in 1 and 3 day-old *Ilp2^1^ gd2HF* flies. (F) Secreted and remaining Ilp2HF content from isolated *Ilp2^1^ gd2HF* heads that were incubated in 3 mM or 100 mM KCl. (G) Oral glucose-stimulated insulin secretion and clearance in 3 day-old *Ilp2^1^ gd2HF* flies, measured by insulin ELISA. Circulating Ilp2HF was measured at 24 hours of fasting, or at 0, 30, 60, and 90 minutes after feeding with 2 M glucose for 30 minutes. In all figures, center values are averages, error bars represent the standard deviation, and two-tailed *t*-tests were used to generate *p* values. * indicates *p*<0.05, ** *p*<0.01, and *** *p*<0.001. N.S. indicates statistically not significant.

To investigate the physiological regulation of *in vivo* Ilp2 levels, we introduced a single copy of the *gd2HF* genomic rescue fragment by site-directed insertion into *Ilp2* null mutants (hereafter *Ilp2^1^ gd2HF*), thereby replacing endogenous *Ilp2* with *Ilp2HF* in the genome. Immunostaining revealed Ilp2HF protein was restricted to adult IPCs of the pars intercerebralis without detectable ectopic expression in *Ilp2^1^ gd2HF* brains (Figure 1E). Circulating trehalose levels were indistinguishable in *Ilp2^1^ gd2HF* adults and controls (Figure 2A). Quantitative reverse-transcriptase polymerase chain reaction (qPCR) revealed that *Ilp2* mRNA levels in *Ilp2^1^ gd2HF* adults and controls were significantly reduced in 3 day-old flies compared to 1 day-old flies (Figure 2B). Thus, *in vivo* regulation of the *gd2HF* genomic rescue fragment recapitulates that of native *Ilp2* and produces the same physiological responses. To quantify total and circulating Ilp2HF in adult flies we developed an ELISA based on commercially available monoclonal antibodies and peptide standards harboring both HA- and FLAG- epitope tags (Figure 2C). This assay detected signal in sample volumes of 1 µl in a standard range of 40 pM to 4 nM (Figure 2C; Materials and Methods). In contrast to mRNA levels, total Ilp2HF content of 1 and 3 day-old homozygous *Ilp2^1^ gd2HF* adults did not change (Figure 2D), demonstrating that changes of *Ilp2* mRNA levels do not strictly correlate with changes in total protein levels. The average circulating Ilp2HF concentration in hemolymph from 1 day-old homozygous *Ilp2^1^ gd2HF* adults was 100 pM and increased to 350 pM in 3 day-old adults (Figure 2E), demonstrating further that levels of secreted Ilp2HF protein in hemolymph from adult flies are regulated independently of total Ilp2HF content. Based on an estimated adult hemolymph volume of 80 nanoliter [16], we determined that the total circulating Ilp2HF rises from 0.05 pg in 1 day-old adult flies to 0.22 pg in 3 day-old adult flies. Thus, we calculate that only 0.1% of total Ilp2HF circulates in the hemolymph of 1 day-old flies, increasing to 0.35% of total Ilp2HF content in 3 day-old flies (Figure 2E). Together these results indicate that a small fraction of total Ilp2HF in IPCs is secreted into the hemolymph *in vivo*, and demonstrate that our ELISA method permits assessment of physiological regulation of insulin production and secretion in flies.

To further assess the fraction of Ilp2 secreted upon stimulation of IPCs, we isolated heads from 3 day-old homozygous *Ilp2^1^ gd2HF* flies, and stimulated them with 100 mM KCl, as previously reported [7]. Under physiological conditions in 3 mM KCl adult hemolymph-like solution (AHLS), 0.2 pg of Ilp2HF (per head) was secreted. This increased 7-fold to 1.4 pg of Ilp2HF (per head) upon stimulation with 100 mM KCl AHLS (Figure 2F). Similar to results *in vivo*, only 0.6% of the total Ilp2 content in heads was secreted in physiological AHLS and the fractional secretion increased to 2.8% of the total when heads were stimulated with 100 mM KCl, comparable to the fraction of insulin secreted from stimulated rat islets [17]. These results emphasize that only a small fraction of total Ilp2 content of IPCs is secreted, even after maximal depolarization induced by 100 mM KCl.

To assess if nutrient availability acutely regulates insulin secretion and circulating levels in hemolymph, as indicated by prior studies [7], we also measured Ilp2HF in fasted and re-fed adult flies. In 3-day old flies fasted for 24 hours then re-fed for 30 minutes, circulating Ilp2HF concentration peaked then declined (Figure 2G), a pattern and timing strikingly similar to that observed in fasted and re-fed humans [18]. Thus, our assays provided measures of systemic insulin levels in flies on a time scale comparable to *in vivo* measures of mammalian insulin dynamics.

### IPC regulators of insulin production and secretion

**Figure 3:**
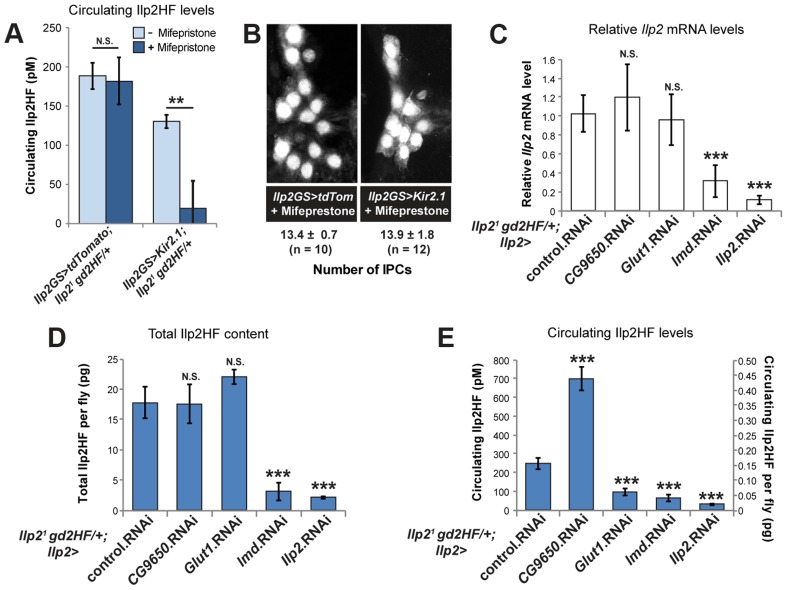
IPC-specific regulators of circulating Ilp2HF. (A) Circulating hemolymph Ilp2HF levels in heterozygous *Ilp2^1^ gd2HF* flies expressing control *tdTomato* or *Kir2.1* by *Ilp2-GeneSwitch (Ilp2GS)* for 2 days with or without Mifepristone feeding. (B) Quantification of IPC cell number in flies expressing control *tdTomato* or *Kir2.1* by *Ilp2-GeneSwitch (Ilp2GS)* for 2 days with Mifepristone feeding. IPCs were marked by *dilp215-1-HStinger*. (C) *Ilp2* mRNA levels in heterozygous *Ilp2^1^ gd2HF* flies with IPC-specific RNAi knockdown of *CG9650*, *Glut1*, *lmd*, and *Ilp2* genes or control mCherry RNAi. (D) Total Ilp2HF protein content in heterozygous *Ilp2^1^ gd2HF* flies with IPC-specific RNAi knockdown of *CG9650*, *Glut1*, *lmd*, and *Ilp2* genes or control mCherry RNAi. (E) Circulating Ilp2HF concentration (pM) or content per fly (pg) in heterozygous *Ilp2^1^ gd2HF* flies with IPC-specific RNAi knockdown of *CG9650*, *Glut1*, *lmd*, and *Ilp2* genes or control mCherry RNAi. In all figures, center values are averages, error bars represent the standard deviation, and two-tailed *t*-tests were used to generate *p* values. * indicates *p*<0.05, ** *p*<0.01, and *** *p*<0.001. N.S. indicates statistically not significant.

IPCs are neurons whose stimulation by glucose evokes electrical responses [19], [20]. Modulating IPC activity is thought to affect insulin release, but changes in circulating insulin levels have not been demonstrated. *Kir2.1* encodes an inward-rectifying potassium channel that has been used to silence electrical activity of *Drosophila* neurons and neuroendocrine cells [21]. We used ‘Geneswitch’ GAL4 to express *Kir2.1* in adult IPCs [22], which permits mifepristone-dependent conditional gene expression, and minimizes the effects of insulin perturbation during animal growth and development. In control *Ilp2^1^ gd2HF* heterozygous flies, circulating Ilp2HF levels were not affected by mifepristone feeding, and were maintained near 200 pM (Figure 3A), about half of the circulating Ilp2HF level in 3 day-old *Ilp2^1^ gd2HF* homozygous flies, as expected. In subsequent experiments, Ilp2HF levels in *Ilp2^1^ gd2HF* heterozygous flies were measured. We found that *Kir2.1* expression induced by mifepristone feeding in adult IPCs significantly reduced hemolymph Ilp2HF concentration without affecting cell number (Figure 3A and B). These results support the postulated role of ion channel activity in regulating insulin secretion [7], and provide direct evidence that ion channel function may couple IPC activation to circulating insulin levels.

Unlike larval IPCs, adult IPCs are glucose responsive [20]. In humans, GLUT1 is a major glucose transporter of pancreatic β-cells. To further assess carbohydrate sensing in adult IPCs, we used RNAi in the IPCs to knockdown expression of the type-1 glucose transporter *Glut1* [23], a gene not previously shown to regulate IPC function. In control experiments, we observed that RNAi knockdown of *Ilp2* significantly reduced *Ilp2* mRNA expression, total Ilp2HF content in flies, and circulating Ilp2HF levels (Figures 3C–E). Knockdown of *Glut1* in IPCs severely reduced circulating Ilp2HF levels, but had no detectable effect on *Ilp2* mRNA levels or total Ilp2HF content in flies (Figures 3C–E). Together, these data suggest that *Glut1* in *Drosophila* IPCs is a conserved regulator of *in vivo* insulin secretion.

To identify uncharacterized regulators of insulin expression, production, and secretion in *Drosophila* IPCs, we performed loss-of-function analysis of fly genes corresponding to GWAS candidate genes for T2DM [2], [24]. *Glis3* was recently shown to be required for insulin expression in mouse islet β-cells [25]. Knockdown of *lmd* (orthologue of *Glis3*) in IPCs severely reduced circulating Ilp2HF levels, total Ilp2HF content, and *Ilp2* mRNA expression (Figures 3C–E), suggesting that *lmd* may regulate Ilp2 expression, similar to the role of rodent *Glis3* in regulating *Ins* expression. *BCL11A* has been associated with type 2 diabetes mellitus [24], but prior work has not linked *BCL11A* to insulin regulation in mammals. In contrast to *lmd*, knockdown of *CG9650* (orthologue of human *BCL11A*) increased circulating Ilp2HF levels without affecting *Ilp2* mRNA levels or total Ilp2HF content in flies (Figures 3C–E), suggesting that *CG9650* may regulate Ilp2HF levels post-translationally.

### Enhanced insulin secretion from impaired peripheral insulin signaling in *Drosophila*

**Figure 4:**
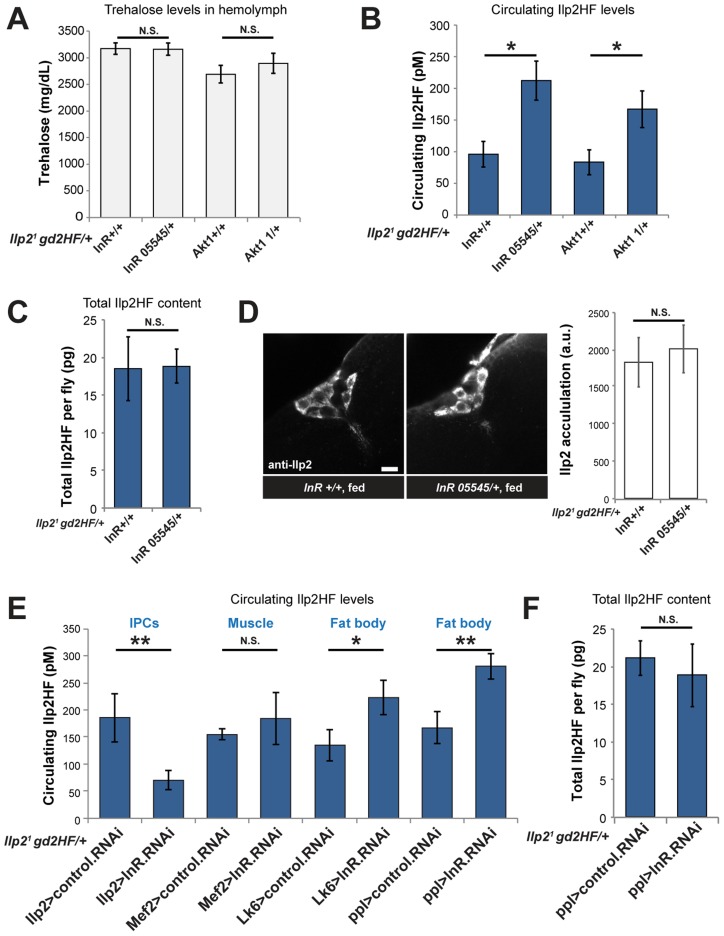
Enhanced insulin secretion from impaired peripheral insulin signaling in *Drosophila*. (A) Circulating trehalose levels in *InR* or *Akt1* heterozygous mutants and sibling wild type control flies. (B) Circulating Ilp2HF levels in *InR* or *Akt1* heterozygous mutants and sibling wild type control flies. (C) Total Ilp2HF amounts in *InR* heterozygous mutants and sibling wild type control flies. (D) Representative image of Ilp2 immunofluorescence in IPCs of *InR* heterozygous mutants and sibling wild type control flies, and average mean signal intensity quantified from summed z-projections (n = 9). Scale bar is 10 µm. (E) Circulating Ilp2HF levels in flies with tissue-specific RNAi knockdown of *InR*. *InR* was knocked down in IPCs (*Ilp2*>), muscle (*Mef*2>), or adult fat body (*Lk6>* and *ppl*>) tissues. (F) Total Ilp2HF content in flies with adult fat body specific RNAi knockdown of *InR* using *ppl-GAL4*. In all figures, center values are averages, error bars represent the standard deviation, and two-tailed *t*-tests were used to generate *p* values. * indicates *p*<0.05, ** *p*<0.01, and *** *p*<0.001. N.S. indicates statistically not significant.

In mammals, genetic or acquired pathological insulin resistance provokes adaptive responses in β-cells, including enhanced insulin secretion [26], but it was not known if such facultative responses were conserved in *Drosophila.* Adult flies heterozygous for the *InR^05545^* mutant allele do not have detectable growth [27] or trehalose phenotypes (Figure 4A). Remarkably, we found that circulating Ilp2HF concentration was doubled in *InR^05545^* heterozygotes (Figure 4B). We observed similar phenotypes in flies heterozygous for a loss-of-function mutation in *Akt1* (Figures 4A and 4B), which encodes an essential conserved regulator of insulin signaling [28]. To test whether elevated hemolymph Ilp2HF levels in *InR^05545^* heterozygous flies derived from increased production or increased secretion, we measured total Ilp2HF content. Ilp2HF content was identical in *InR^05545^* heterozygous flies and controls (Figure 4C), indicating that hyperinsulimia in *InR^05545^* heterozygotes results from enhanced insulin secretion, not from enhanced insulin production. These results are reminiscent of adaptive phenotypes noted in mice harboring heterozygous mutations in genes encoding insulin receptor or other insulin signaling regulators [29], [30]. The two-fold increase in circulating Ilp2HF in *InR^05545^* heterozygotes represents only a minor fraction of the total Ilp2HF content; thus we asked whether this subtle difference could be detected using previously established methods [7]. We could not detect differences in Ilp2 accumulation in IPCs from *InR^05545^* heterozygotes and control flies by immunofluorescence (Figure 4D), suggesting that quantification of *Drosophila* insulin by the Ilp2HF system permits the discovery and characterization of phenotypes not detected by semi-quantitative assays of Ilp2 secretion.

To identify the tissue-specific basis of the enhanced insulin secretion phenotypes in heterozygous *InR* mutants, we systematically knocked down *InR* expression using RNAi in adult IPCs, muscle, or in fat body, an organ with functions orthologous to the liver. *InR* knockdown in muscle using *Mef2-GAL4* did not detectably alter Ilp2HF levels (Figure 4E), reminiscent of normal serum insulin levels observed in muscle-specific insulin receptor knockout (MIRKO) mice [31]. *InR* knockdown in IPCs using *Ilp215-1-GAL4* decreased circulating Ilp2HF levels (Figure 4E), reminiscent of insulin defects found in pancreatic β cell-specific insulin receptor knockout (BIRKO) mice [32]. In contrast, increased circulating Ilp2HF levels were evoked by RNAi-mediated *InR* knockdown in the adult fat body using *Lk6-GAL4* or *ppl-GAL4* (Figure 4E) without detectable effects on total Ilp2HF content in fat body-specific *InR* knockdown using *ppl-GAL4* (Figure 4F). RNAi-mediated *InR* knockdown in fat body was confirmed by qPCR of fat body cDNAs (Figure S3). These results suggest that insulin secretion, not production, from IPCs is regulated by impaired insulin signaling in fat body. Thus, similar to mice with conditional insulin receptor loss in liver (LIRKO) [33], targeted impairment of insulin signaling in *Drosophila* fat body produced enhanced insulin secretion from IPCs.

## Discussion

*In vivo* measures of circulating insulin and other peptide hormones in organisms with powerful experimental advantages, like *Drosophila*, could transform the scope of physiological and genetic approaches possible in these systems, and advance their use for metabolic and genomic studies. Active insulin is produced from multiple post-translational processing steps, including proteolytic cleavage and extensive disulfide bonding, and modification of a single amino acid in insulin protein can significantly alter or eliminate its hormone activity. Thus, despite intensive efforts, labeling of insulin with useful peptide epitopes while preserving *in vivo* hormone function has remained a challenge. We exploited quantitative synthetic lethality tests in flies to screen multiple modifications in the Ilp2 protein, and found that Ilp2 tolerated epitope insertions only at specific locations while preserving bioactivity, specifically the HA-epitope at the B-chain carboxy-terminus and the FLAG-epitope at the A-chain amino-terminus. While structural analysis for Ilp2 is not available, to our knowledge, the structure of the related insulin-like peptide Ilp5 has been reported [6], revealing a disordered B-chain carboxy-terminus adjacent to the A-chain amino-terminus. To the extent that similar features may be found in Ilp2, we speculate that this structural feature may be permissive for Ilp2 epitope tagging while preserving function. If so, epitope-tagging methods described here may be used to quantify and investigate function of other processed circulating peptide hormones in *Drosophila*, or other species. We also found that Ilp2HF bioactivity is impaired by introduction of an “Akita” missense mutation, analogous to mutations previously shown to disrupt post-translational insulin processing in rodents, and in humans with dominant mutant proinsulin syndrome [14], [15]. This raises the likelihood that conserved mechanisms may underlie prepro-Ilp2 processing and folding in *Drosophila* IPCs, and that the *Ilp2–3,5 gd2HF.C119Y* line may provide a useful model for studies of protein-folding in *Drosophila*.

Our studies revealed that *Drosophila* insulin expression, production, and secretion are dynamic and independently regulated in IPCs. By contrast, intracellular immunofluorescence methods that infer IPC secretion responses do not discriminate between insulin expression, production, and secretion. Moreover we also found that, upon IPC stimulation, only a small fraction of the total Ilp2 in IPCs is secreted *in vivo* and *in vitro*. Based on synthesized peptide standards, we found the circulating Ilp2HF concentration increases from 100 pM in 1 day-old flies to 350 pM in 3 day-old flies without a change of total Ilp2 content during this period. Although Ilp2 affinity for the *Drosophila* insulin receptor has not been reported, competitive binding studies of purified Ilp5 revealed a *K_d_* of 350–760 pM [6], consistent with our *in vivo* findings. Since distinct Ilps produced in IPCs may be co-released, Ilp2 levels may indirectly reflect release of Ilp3 and Ilp5 from IPCs. While the basis for enhanced Ilp2HF secretion in 3 day old flies is not yet known, feeding behavior may change over this period and underlie this effect. Alternatively, stimulus-secretion coupling mechanisms in IPCs may mature in the first 3 days. Both possibilities have been previously observed during the postnatal weaning and maturation period in mammals. Thus, a scalable and highly sensitive method of measuring insulin content and secretion should enable a new class of physiological studies in *Drosophila*, permitting genetic dissection of feeding behaviors and diet effects on insulin signaling.

Our system revealed molecular and cellular mechanisms for two fly orthologues of T2DM risk genes in regulating systemic insulin levels. *Glis3* was recently shown to be required for insulin expression in mouse islet β-cells [25]. Consistent with this finding, we found that IPC knockdown of *lmd*, a fly orthologue of human *GLIS3*, reduced *Ilp2* mRNA and total Ilp2HF protein levels, suggesting conserved mechanisms regulating insulin expression. In contrast, we found that *CG9650* knockdown in IPCs increased circulating Ilp2HF levels, without affecting Ilp2 expression or production. Thus, the product of *CG9650* likely regulates circulating insulin levels at a post-translational step. Prior studies suggested that *CG9650* encodes a transcription factor with roles in axon guidance, Notch signaling and oxidative stress responses [34]–[36], but did not identify roles in insulin processing or secretion. Likewise *BCL11A*, a human orthologue of *CG9650*, has been associated with type 2 diabetes mellitus, but prior work has not linked *BCL11A* to insulin regulation in mammals. In addition, our system now permits further studies of circulating signals or neurotransmitters thought to regulate insulin secretion by IPCs, including Ilp6, Unpaired 2, and serotonin [9], [37], [38]. Thus, the ability of our system to measure insulin production and secretion permits mechanistic evaluation and linkage of candidate human diabetes susceptibility genes to roles in insulin expression, post-translational processing, or secretion.

Using our system, we also detected adaptive enhancement of insulin secretion in flies with heterozygous *InR* or *Akt1* mutations. These phenotypes are similar to those reported in mice with *IR* or *IRS* deficiency [29], [30], in which impaired insulin signaling in peripheral tissues promotes a ‘pre-diabetic’ condition with adaptive hyperinsulinemia compensating for systemic insulin resistance while maintaining normoglycemia. The detection and quantification of haploinsufficiency phenotypes in heterozygous *InR* or *Akt1* mutants suggests that genetic screens using deficiency lines could identify novel regulators of insulin production and secretion. We also observed changes in circulating Ilp2 levels after specific knockdown of the insulin receptor in *Drosophila* fat body or IPCs, but not in muscle. These results are consistent with prior reports that fat body signals might regulate IPCs [7], and suggest a role for fat body insulin-signaling in feedback regulation of systemic insulin levels in *Drosophila.* The changes in circulating Ilp2HF levels after *InR* knockdown in fly fat body, IPCs or muscle, were remarkably similar to changes in serum insulin observed after tissue-specific knock-out of insulin receptor in mouse liver, pancreatic β-cells or muscle [31]–[33], the so-called LIRKO, BIRKO and MIRKO mice. Thus, integrated analyses permitted by our assays revealed that mechanisms governing facultative adaptation to pathological states like impaired insulin signaling in multiple target organs are maintained from insects to mammals. We speculate that *in vivo* Ilp2HF quantification in *Drosophila* should be useful to identify conserved regulators of insulin expression, secretion and responsiveness relevant to human health and diseases.

## Materials and Methods

### *Drosophila* strains

*y^1^ w^1118^* (Bloomington stock ID #6598), *Ilp2^1^* (#30881), *Df(3L)Ilp2–3,Ilp5^3^/TM3* (#30889), *InR^05545^/TM3* (#1161), *Act5C-GAL4/CyO* (#4414), *Mef2-GAL4* (#27390), *Lk6-GAL4* (#8614), *UAS-CD4-tdTomato* (#35841), *UAS-Kir2.1-eGFP* (#6596), *UAS-mCherry.RNAi* (#35785; used as a control RNAi), *UAS-InR.RNAi* (#31594), *UAS-Ilp2.RNAi* (#31068), *UAS-lmd.RNAi* (#42871), *UAS-CG9650.RNAi* (#26713), and *UAS-Glut1.RNAi* (#40904) used in this study were obtained from Bloomington Stock Center. *Drosophila* orthologues for human genes were identified by Ensemble release 73. *Ilp215-1-GAL4* used in this study is made from pIlp215-1-GAL4 construct (See below), and its adult expression is restricted in IPCs. *Akt1^1^/TM3* was provided by Dr. Clive Wilson (University of Oxford). UAS-FLAG-dilp2 [12] was provided by Dr. Matt Scott (Stanford University). *ppl-GAL4* [39] was provided by Dr. Michael Pankratz (Universität Bonn). *Ilp2-GeneSwitch* was provided by Dr. Yih-Woei C. Fridell (University of Connecticut). *dilp215-1-HStinger* was previously described [40]. In all experiments, animals were either fed on cornmeal/dextrose/yeast food *ad libitum*, fasted on 1% agar only food, or re-fed on 2M glucose in 1% agar with 0.05% bromophenol blue for oral glucose-stimulated insulin secretion experiments at 22°C. Standard *Drosophila* cornmeal/dextrose/yeast food was prepared with the recipe: 1% (w/v) agar, 5% (w/v) cornmeal, 10% (w/v) dextrose, and 2.5% (w/v) baker's yeast. Please note that 10% (w/v) dextrose is about 555 mM. Mifeprestone (Sigma-Aldrich M8046) was added at 0.2 mM when needed.

### Generation of transgenic lines

pIlp215-1-GAL4 was generated by subcloning the 541 bp sequence upstream of the *Ilp2* transcription start site [4] into pPTGAL. pUAST-Ilp2 was generated by subcloning 705 bp EcoR1-Xho1 fragment from DGC clone GH11579. pUAST-Ilp2HF was generated by PCR-based site-directed mutagenesis to add 5′-TAT CCA TAT GAT GTT CCT GAC TAT GCT-3′ (encoding the amino acids YPYDVPDYA) sequence after the end of Ilp2 B-chain and 5′-GAT TAT AAG GAC GAC GAT GAC AAG-3′ (encoding the amino acids DYKDDDDK) sequence before the beginning of Ilp2 A-chain (See Figure 1A). *P*-element mediated germline transformations were carried out to generate *Ilp215-1-GAL4*, *UAS-Ilp2*, and *UAS-Ilp2HF* transgenic lines. 2413 bp genomic *Ilp2* region was amplified from *y^1^ w^1118^* genomic DNA using 5′-CCGAGAATTCACACTTGGCCAACACACACACATTCATTA-3′ and 5′-ACTGTCTAGAATTGGCCAACTTGATTGGTAATGAAACGG-3′ primers and subcloned to EcoR1 and Xba1 sites on pBDP2 (a modified version of pBDP with EcoR1, Xba1, and Not1 cloning sites) [41] to generate pBDP2-gd2. pBDP2-gd2HF was generated by replacing *Ilp2* coding region in pBPD2-gd2 with *Ilp2HF* ORF. pBDP2-gd2HF.C119Y was generated by PCR-based site-directed mutagenesis to change from 5′-TGCTGCAA-3′ to 5′-TGTTATAA-3′. *phiC31* integrase-mediated germline transformations were carried out to generate *gd2(attP2)*, *gd2HF(attP2)*, and *gd2HF.C119Y(attP2)* transgenic lines using Bloomington stock #25710. *gd2(attP2)*, *gd2HF(attP2)*, or *gd2HF.C119Y(attP2)* transgene was recombined to *Df(3L)Ilp2–3, Ilp5* ^3^ mutant backgrounds to assess phenotypic rescue of *Ilp2–3, 5* deficiency mutant. To replace endogenous *Ilp2* gene with *gd2HF(attP2)* in the genome, the *gd2HF(attP2)* transgene was recombined into *Ilp2^1^* mutant chromosome to generate the *y^1^ w^1118^; Ilp2^1^ gd2HF(attP2)* strain which was used to measure the circulating Ilp2HF in hemolymph. Please note that the *y^1^ w^1118^; Ilp2^1^ gd2HF(attP2)* strain is homozygous for *gd2HF*, and their circulating llp2HF levels are 300–400 pM (Figure 2E). To express *Kir2.1* in insulin producing cells conditionally, the *Ilp2-GeneSwitch/CyO; Ilp2^1^ gd2HF(attP2) dilp215-1-HStinger* strain was crossed to flies harboring transgene encoding *UAS-Kir2.1-eGFP*, and appropriate progeny were fed 200 µM Mifeprestone or vehicle (ethanol) in cornmeal/dextrose/yeast food for 48 hours. The progeny carry only one copy of *gd2HF*, and their circulating Ilp2HF levels are 100–200 pM (Figure 3A). To knockdown genes in adult IPCs and measure Ilp2HF in hemolymph, TRiP RNAi lines were crossed to the *UAS-Dcr-2.D; Ilp2^1^ gd2HF(attP2) Ilp215-1-GAL4* strain. To knockdown genes in adult muscles and measure circulating Ilp2HF in hemolymph, TRiP RNAi lines were crossed to the *UAS-Dcr-2.D; Ilp2^1^ gd2HF(attP2) Mef2-GAL4* strain. To knockdown genes in adult fat body tissues and measure circulating Ilp2HF in hemolymph, TRiP RNAi lines were crossed to either the *UAS-Dcr-2.D; Ilp2^1^ gd2HF(attP2) Lk6-GAL4* or the *ppl-GAL4 UAS-Dcr-2.D; Ilp2^1^ gd2HF(attP2)* strain. Progeny from these crosses carry only one copy of *gd2HF*, and their circulating Ilp2HF level are 100–200 pM (Figure 3E and Figure 4B and E).

### Wing length measurement

One wing per female fly was dissected in isopropanol, mounted in Canada balsam:Methyl salicylate (4∶1) on a slide, and heated on 65°C hot plate for 1 hour to harden the mounting media. The distance between the distal end of the L3 wing vein and the posterior end of wing hinge was measured using AxioVision software. 5 wing spans were measured per genotype, and statistical differences between genotypes were determined with a two-tailed Student's *t*-test. The results are presented as the mean ± standard deviation.

### Immunohistochemistry

Immunostaining of adult brains was performed as described [40] with modifications: Affinity purified rabbit polyclonal anti-Dilp2 antibody (0.5 µg/ml), mouse monoclonal anti-FLAG M2 antibody (1 µg/ml; Sigma-Aldrich F1804), Rat monoclonal anti-HA 3F10 antibody (0.1 µg/ml; Roche 1867423), and Alexa Fluor 488, 547, and 647 secondary antibodies (2 µg/ml; Life Technologies) were diluted and incubated in PBS with 0.3% Triton-X100. Confocal laser scanning microscope images were obtained using Leica TCS SP5 or SP8. Accumulation Ilp2 in IPCs was quantified as described previously [7]. Adult brains were dissected and stained for immunofluorescence as described above, and mounted with the IPCs oriented towards the coverslip. Confocal imaging parameters were optimized such that images of all samples could be acquired within the dynamic range of constant laser and scan settings. Confocal Z stacks of the IPCs were acquired with a step size of 1 µm. For quantification, image stacks were summed and the mean-pixel intensity of a region of interest (ROI) containing the entire IPC cluster was measured and subjected to background subtraction using an ROI drawn adjacent to the cell cluster. Average mean pixel intensity of IPCs across biological replicate brains for each condition is expressed in arbitrary units (a.u.).

### Hemolymph sample collection using spin columns

In all assays, three separate samples per specific fly group or condition were collected. Unless otherwise noted, 3-day-old male flies fed *ad libitum* were used in all experiments. We avoided using female flies due to possible feeding behavior changes in virgin and mated females [42], and larger observed standard deviations in all metabolic assays we used. All flies were transferred to vials with fresh food 24 hours prior to hemolymph collection to ensure similar nutritional conditions except fasted groups which were maintained on vials with 1% agar for 24 hours prior to the collection. In acute re-feeding and re-fasting experiments, fasted flies were placed on 2M glucose in 1% agar with 0.05% bromophenol blue for 30 minutes, then re-fasted flies. Because not all 24-hour fasted flies commence feeding on 2M glucose, only flies with visibly ingested blue food coloring in their gut were selected for hemolymph sampling. To elute hemolymph, sixty male flies per group were placed in a modified Zymo-Spin IIIC column (Zymo Research Corporation C1006) in which DNA-binding filter were removed and thoroughly washed with water. The column containing male flies was centrifuged twice at 9,000 g for 5 minutes at 4°C. This yielded approximately 1.5 µl hemolymph, which was used for either trehalose assays or ELISA.

### Extraction of protein content from adult flies

A single fly was placed in a 1.5 mL centrifuge tube with 100 µl of PBS containing 1% Triton X-100. Four samples were prepared for each genotype. The samples were ground using a pestle and cordless motor (VWR 47747-370), and lysed at room temperature for 30 minutes on a rotary shaker. The lysed samples were centrifuged at 21,000 g for 5 minutes at room temperature and 50 µl supernatant from the centrifuged samples were used for ELISA.

### *In vitro* Ilp2HF secretion assay from isolated heads

Adult hemolyph-like saline (AHLS) was prepared with the recipe: 2 mM CaCl_2_, 3 mM KCl, 8.2 mM MgCl_2_, 108 mM NaCl, 4 mM NaHCO_3_, 1 mM NaH_2_PO_4_, 3 mM Glucose, and 2% bovine serum albumin. Heads of 3 day-old *Ilp2^1^ gd2HF* males were carefully separated from bodies in AHLS to maintain foregut and crop. 15 isolated heads were transferred to a centrifuge tube containing 100 µl of AHLS, and allowed to recover for 1 hour at room temperature. Three samples per condition were prepared. The samples were washed 3 times with AHLS, and incubated in 100 µl of AHLS containing either 3 mM or 100 mM KCl for 30 minutes. 100 µl of the incubated AHLS from the samples was saved and 50 µl was used for ELISA. To measure total content, 150 µl of PBS containing 1% Triton X-100 was added to the remaining heads in the tubes, and the samples were ground using a pestle and cordless motor. After 30 minutes lysis at room temperature, the samples were centrifuged at 21,000 g for 5 minutes. 10 µl supernatant from the centrifuged samples was diluted in 90 µl PBS, and 50 µl of the diluted sample were used for ELISA.

### Ilp2HF ELISA

We coated wells in Nunc-Immuno modules (Thermo Scientific 468667) with 100 µl of anti-FLAG antibody (Sigma-Aldrich F1804) diluted in 0.2 M sodium carbonate/bicarbonate buffer (pH9.4) to a final concentration of 2.5 µg/ml, then incubated for 16 hours at 4°C. The plate was washed twice with PBS containing 0.2% Tween 20 (PBTw0.2), then coated with 350 µl of PBS containing 2% bovine serum albumin (Fisher Scientific BP1600) for 16 hours at 4°C. The plate was washed three times with PBTw0.2. For circulating Ilp2HF measurement, 1 µl of hemolymph or 1 µl of FLAG(GS)HA peptide standards (DYKDDDDKGGGGSYPYDVPDYAamide, 2412 daltons: LifeTein LLC) at 0.1–10 pg/µl was mixed with 50 µl of PBS containing 1% Triton X-100 and 5 ng/ml anti-HA-Peroxidase 3F10 antibody (Roche 12013819001), vortexed, centrifuged briefly, and transferred to wells on the plate. For total Ilp2HF content measurement, 50 µl of supernatant from single fly lysate or 50 µl of FLAG(GS)HA peptide standards at 5–500 fg/µl was mixed with 50 µl of PBS containing 1% Triton X-100 and 5 ng/ml anti-HA-Peroxidase 3F10 antibody, vortexed, centrifuged briefly, and transferred to wells on the plate. For samples derived from *in vitro* head assays, 50 µl of collected media or diluted head lysate was used. The wells were sealed with an adhesive film (Thermo Scientific 232698), and incubated in a humidity chamber for 16 hours at 4°C. We removed the samples by aspirating, and washed the wells six times with PBTw0.2. 100 µl of 1-Step Ultra TMB ELISA Substrate (Thermo Scientific 34029) was added to each well and incubated on a rotary shaker for 30 minutes at room temperature. The reaction was stopped by adding 100 µl of 2 M sulfuric acid, and the absorbance at 450 nm (A_450_) was immediately measured on a SpectraMax M5 (Molecular Devices). To convert concentration to mass in a given volume, we used a molecular weight of 7829 daltons for mature Ilp2HF protein.

### Trehalose assay

1 µl eluted sample from the centrifuged flies was diluted in 9 µl PBS, vortexed, centrifuged briefly, and heated at 70°C for 5 minutes to inactivate endogenous Trehalase. 2 µl of the heated sample was added to 200 µl of Glucose Hexokinase Reagent (Thermo Scientific TR15421) with or without Porcine Kidney Trehalase (1∶1000; Sigma-Aldrich T8778-5UN), incubated at 37°C for 16 hours, and the absorbance at 340 nm (A_340_) was measured on a SpectraMax M5. The trehalose concentration in the sample was determined by subtracting the glucose concentration from the total sugar concentration.

### Quantitative RT-PCR

Four female flies per group with three biological replicates were homogenized in 600 µl of TRIzol Reagent (Life Technologies 15596-018), and total RNA was isolated according to the manufacturer's protocol. To isolate total RNA from larval fat body, we dissected fat body tissues from 6 larva per group. Three biological replicates were homogenized in 600 µl of TRIzol Reagent. Total RNA pellet was resuspended in 30 µl of water. 1 µg of total RNA was treated with DNAse I, heat-inactivated, and reverse transcribed in 10 µl reaction using High Capacity cDNA Reverse Transcription Kit (Applied Biosystems 4368814). 1.5 µl of cDNA was used in a final volume of 15 µl for quantitative PCR reaction (Solaris qPCR Low ROX Master Mix, Thermo Scientific AB-4352/C), and PCR amplification was detected by 7500 Real Time PCR system (Applied Biosystems). Relative expression levels of *Ilp2* or *Ilp2HF* were determined by Applied Biosystems Taqman probe for *Ilp2* (Dm01822534_g1). Relative expression levels of *InR* were determined by Applied Biosystems Taqman probe for *InR* (Dm02136224_g1). Applied Biosystems Taqman probe for *Rpl32* (Dm02151827_g1) was used as the internal control to determine relative expression of *Ilp2* and *InR*.

## Supporting Information

Figure S1 Synthetic lethality test of epitope tagged *Ilp2* variants. HA (blue) or FLAG (purple) sequences were inserted at various positions of B or A chain of Ilp2 sequence. At least two independent transgenic lines per each UAS construct were tested for F1 lethality by crossing to *actin5C-GAL4*. The percentage of F1 lethality was determined by comparing the number of F1 progeny with or without *actin5C-GAL4* ectopic tissue driver. * denotes the C119Y point mutation introduced in *UAS-Ilp2HF* transgene. SP denotes the signal peptide. The numbers in parenthesis indicate the number of F1 progeny without *actin5C-GAL4*.
(PDF)

Figure S2 Epitope-tagged Ilp2 rescues the developmental delay in insulin deficient flies. Developmental duration (days) from egg deposition to adult eclosion for female (red bars) or male (blue bars) flies of the indicated genotypes were recorded daily.
(PDF)

Figure S3 *InR* mRNA levels in larval fat body of fat body-specific *InR* knockdown animals. Relative *InR* mRNA levels in isolated larval fat body tissues from larva expressing *ppl-GAL4* driver and the control *mCherry* RNAi or *InR* RNAi, as indicated. Center values are averages, error bars represent the standard deviation, and two-tailed *t*-tests were used to generate *p* values.
(PDF)
