# Supplementary material for: A Genetic Strategy to Measure Circulating Drosophila Insulin Reveals Genes Regulating Insulin Production and Secretion
Source: PLoS Genet. 2014 Aug 7;10(8):e1004555. doi: 10.1371/journal.pgen.1004555 (PMC4125106; doi:10.1371/journal.pgen.1004555)
Supplement: Figure S1 — Synthetic lethality test of epitope tagged Ilp2 variants. HA (blue) or FLAG (purple) sequences were inserted at various positions of B or A chain of Ilp2 sequence. At least two independent transgenic lines per each UAS construct were tested for F1 lethality by crossing to actin5C-GAL4. The percentage of F1 lethality was determined by comparing the number of F1 progeny with or without actin5C-GAL4 ectopic tissue driver. * denotes the C119Y point mutation introduced in UAS-Ilp2HF transgene. SP denotes the signal peptide. The numbers in parenthesis indicate the number of F1 progeny without actin5C-GAL4. (PDF) [file pgen.1004555.s001.pdf]

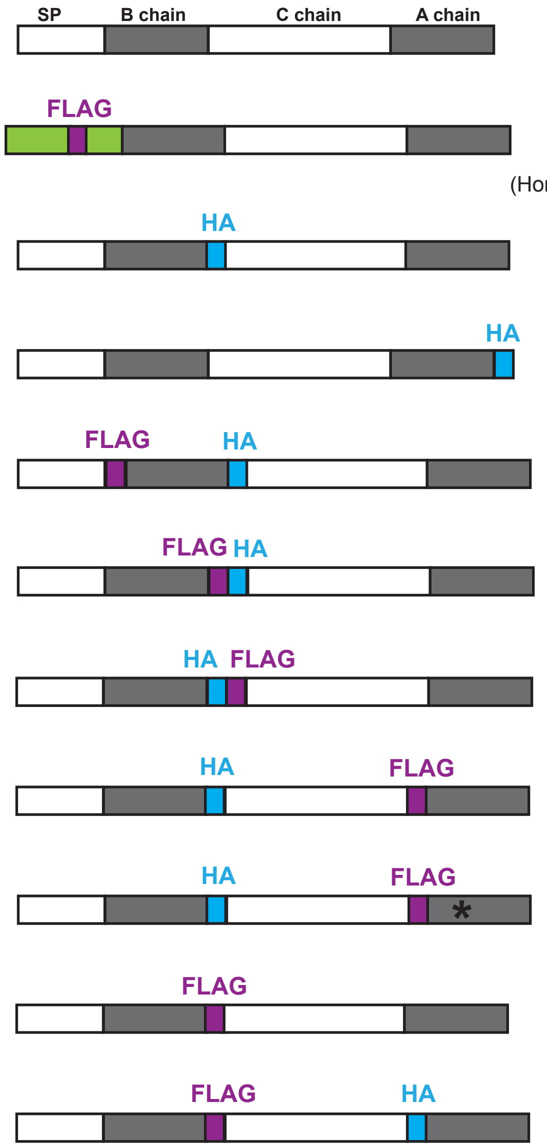

actin5C-GAL4 / CyO X	Lethality of F1 (act5C>UAS)
UAS-Ilp2	100 % (n = 222)
UAS-Dilp2^{F} (Honegger, 2008; Pasco and Léopold, 2012)	38.2 % (n = 87)
UAS-Ilp2-BHA	99.3 % (n = 572)
UAS-Ilp2-AHA	85.4 % (n = 822)
UAS-Ilp2-FLAGBHA	39.4 % (n = 180)
UAS-Ilp2-BFLAGHA	46.4 % (n = 153)
UAS-Ilp2-BHAFLAG	16.3 % (n = 55)
UAS-Ilp2-BHA-FLAGA (**Ilp2HF**)	100 % (n = 125)
UAS-Ilp2-BHA-FLAGA.C119Y	0 % (n = 102)
UAS-Ilp2-BFLAG	55.7 % (n = 460)
UAS-Ilp2-BFLAG-HAA	61.7 % (n = 779)
