# Supplementary figures and images for: A Genetic Strategy to Measure Circulating Drosophila Insulin Reveals Genes Regulating Insulin Production and Secretion
Source: PLoS Genet. 2014 Aug 7;10(8):e1004555. doi: 10.1371/journal.pgen.1004555 (PMC4125106; doi:10.1371/journal.pgen.1004555)

### *Ilp2-3,5* <sup>+/+</sup>

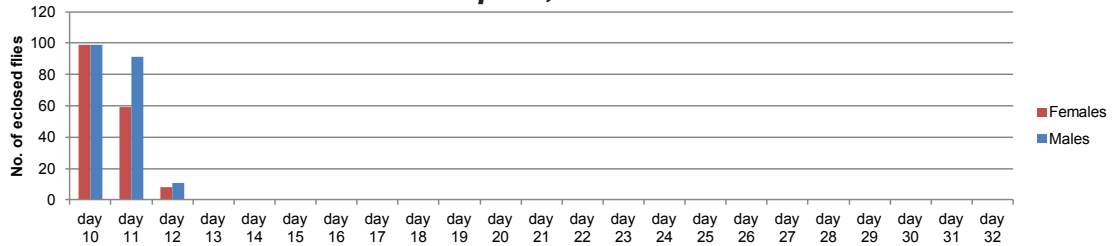

### *Ilp2-3,5*

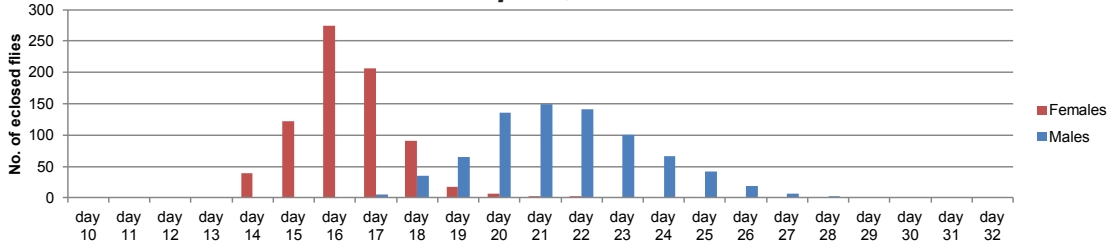

### *Ilp2-3,5* *gd2*

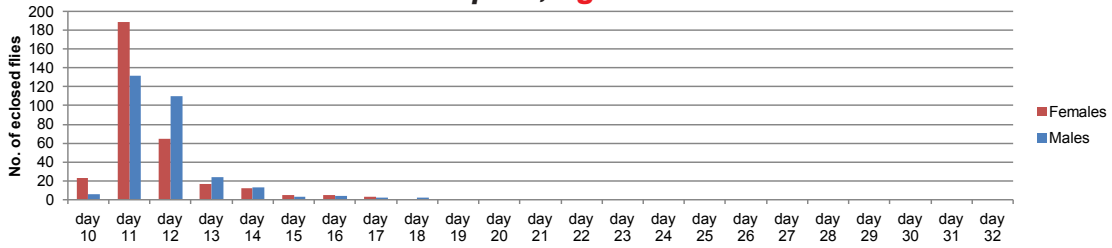

### *Ilp2-3,5* *gd2HF*

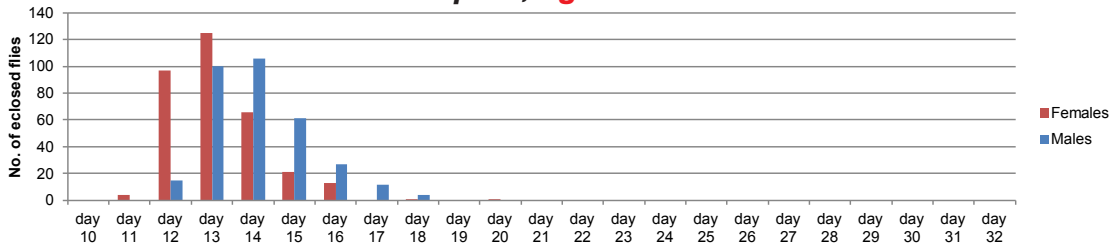

### *Ilp2-3,5* *gd2HF.C119Y*

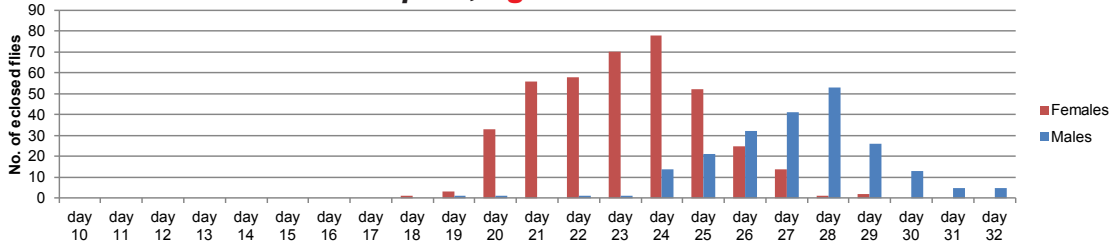

Supplement: Figure S2 — Epitope-tagged Ilp2 rescues the developmental delay in insulin deficient flies. Developmental duration (days) from egg deposition to adult eclosion for female (red bars) or male (blue bars) flies of the indicated genotypes were recorded daily. (PDF) [file pgen.1004555.s002.pdf]

### ***InR* mRNA levels in fat body**

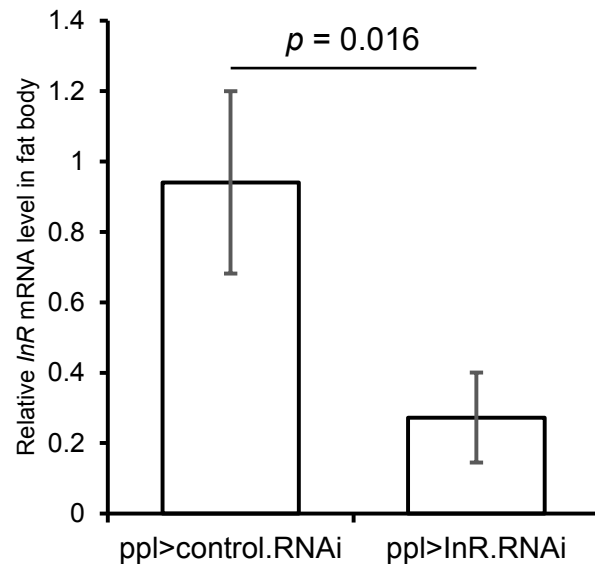

Supplement: Figure S3 — InR mRNA levels in larval fat body of fat body-specific InR knockdown animals. Relative InR mRNA levels in isolated larval fat body tissues from larva expressing ppl-GAL4 driver and the control mCherry RNAi or InR RNAi, as indicated. Center values are averages, error bars represent the standard deviation, and two-tailed t-tests were used to generate p values. (PDF) [file pgen.1004555.s003.pdf]
